# 肺原发性韦格纳肉芽肿1例：附文献综述

**DOI:** 10.3779/j.issn.1009-3419.2021.101.47

**Published:** 2021-12-20

**Authors:** 典 任, 昕 李, 明辉 刘, 京豪 刘, 钢 陈, 春秋 夏, 超翼 贾, 军 陈

**Affiliations:** 300052 天津，天津医科大学总医院肺部肿瘤外科 Department Lung Cancer Surgery, Tianjin Medical University General Hospital, Tianjin 300052, China

**Keywords:** 韦格纳肉芽肿, 肺肿瘤, 经皮肺穿刺, 自身免疫性疾病, 病例报告, Wegener' granulomatosis, Lung neoplasms, Percutaneous lung biopsy, Autoimmune disease, Case report

## Abstract

韦格纳肉芽肿（Wegener’ granulomatosis, WG）是一种病情发展迅速的自身免疫性疾病，可累及肺、肾脏等全身各个器官，未得到早期诊疗的患者死亡率较高。由于WG发病率极低且缺乏特异性影像学特征，常导致临床漏诊、误诊和治疗延误。本文报道1例55岁女性患者，因肺部结节经皮肺穿刺活检确诊为肺原发性WG的诊疗经过，并对相关文献进行了综述。

## Abstract (en)

Wegener' granulomatosis is an autoimmune diseases, often involving the lung and kidney, has a high mortality rate in nontreatment patients. The low incidence and nonspecific features, often lead to misdiagnosis and delayed treatment. This paper reported the diagnosis and treatment of a 55-year-old female patient with primary Wegener' granuloma of the lung diagnosed by percutaneous lung biopsy of pulmonary nodules, and reviews the relevant literature.

韦格纳肉芽肿（Wegener’ granulomatosis, WG），又称为肉芽肿性血管炎（granulomatosis with polyangiitis, GPA），是一种可累及全身各个器官的自身免疫性疾病，其发病率为0.2/10, 000-0.3/10, 000，其中97%的患者是高加索人，2%为黑人，1%为其他种族。由于WG目前尚无特异性确诊手段且较为罕见，临床工作中对该病的误诊率极高，大多数患者预后不良的主要因素是未能早期确诊导致的治疗延误^[1]^。若不及时治疗，患者平均生存时间仅为5个月，82%的患者于1年内死亡，90%的患者2年内死亡^[2]^。因此，早期明确诊断成为改善患者预后的有效途径。本文报道1例以肺占位为首发症状、具有典型WG临床表现的患者的临床诊断过程，并进行文献复习，旨在提高临床医生对该疾病的鉴别诊断水平及应用计算机断层扫描（computed tomography, CT）引导下经皮肺穿刺在WG诊断中的认识。

## 1 临床资料

患者，女，55岁，患者于2014年3月无明显诱因出现后背部疼痛，逐渐发展至肋部疼痛，数天后无明显诱因好转。2周后出现午后潮热，体温最高37.2 oC，每天发作3次左右，每次持续1 h，无特殊处理，发汗后可自行退热，于当地医院就诊，胸片结果考虑肺结核，入结核病专科医院抗结核药物治疗，未有明显好转。血液学检查结果中，除白细胞升高外（最高15.96×10^9^/L；中性粒细胞绝对值12.92×10^9^/L；中性粒细胞比：0.809），真菌及结核相关检查均未见明显异常，肺肿瘤标志物不高。支气管镜检查未见明显肿物，于左上尖支气管行灌洗及刷检，送检标本均未见肿瘤细胞。

患者为排除肿瘤性病变转入我科，入院后很快出现肾脏损害，入院时尿常规：红细胞计数48.0个/μL（参考值0个/μL-30.7个/μL）。10 d后复查尿常规：尿蛋白1+；白细胞2+；潜血3+；尿沉渣：①白细胞：212.6个/μL；②红细胞：366.3个/μL；③上皮细胞：68.3个/μL。查泌尿系彩超双肾、膀胱彩超及腹部彩超均未见明显异常。

因患者无明显诱因相继出现肺部和肾脏的多器官损伤，且进展迅速，符合WG的典型临床表现，查免疫全项：抗中性粒细胞胞浆抗体（antineutrophil cytoplasmic antibodies, ANCA）-C型阳性；ANCA-P型阳性；抗髓过氧化物酶（myeloperoxidase, MPO）抗体 < 20 RU/mL；抗蛋白酶3（proteinase 3, PR3）抗体261.69 RU/mL。

患者表现为呼吸系统及肾脏联合损害，同时ANCA相关指标阳性，目前诊断考虑WG可能性大。但WG的诊断除了需满足相关诊断标准外，还需除外结核、肿瘤等疾病，考虑给予CT引导下经皮肺穿刺明确病理，遂于图 1所示位置行经皮肺穿刺活检。术后病理回报（图 2）：肺组织示慢性炎症，肺泡间隔内可见淋巴细胞、浆细胞及单核细胞浸润，于骨骼肌组织中检见小血管、血管壁纤维素样坏死伴中性粒细胞渗出，血管周围上皮细胞肉芽肿形成，结合临床，考虑Wegener肉芽肿可能，拟转入风湿科继续内科治疗。经皮肺穿刺后术后第2天晚，患者诉无明显诱因出现左腹部疼痛，伴有脐周及左侧腰背部疼痛，查体左腰部叩击痛，全腹无明显压痛反跳痛，无腹肌紧张，给予泌尿系B超及全腹部平扫CT回示左侧肾筋膜血肿（图 3），泌尿外科会诊考虑暂给予止疼等保守治疗，转入风湿科继续内科治疗。经规范治疗，患者于2周后好转出院，之后7年间风湿免疫科反复住院治疗，但治疗效果良好，至今存活。

**图 1:**
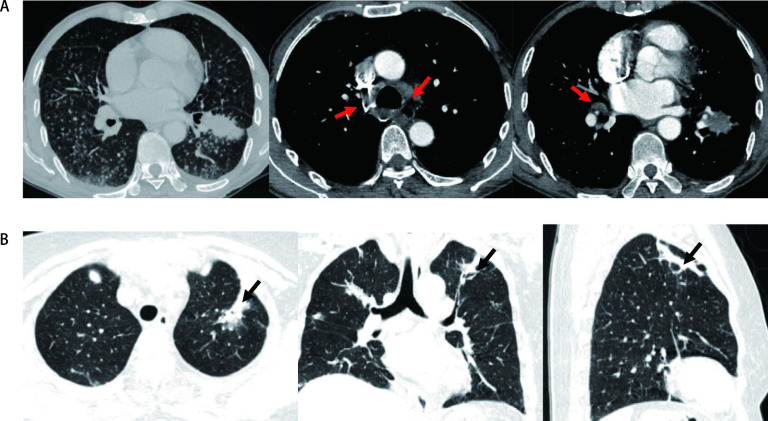
患者胸部CT检查。A：肺部不规则高密度影，伴有纵隔淋巴结肿大；B：于左肺上叶高密度不规则肿物影处行CT引导下经皮肺穿刺活检。红色箭头：肿大的纵隔淋巴结；黑色箭头：经皮肺穿刺活检的穿刺点。 CT scans of the chest. A: The chest CT indicating the irregular high-density shadows of the lung. There were amount of irregular infiltration shadow volume around and mediastinal lymph node enlargement; B: An image-guided percutaneous transthoracic needle biopsy (PTNB) is performed on the mass in the upper lobe of the left lung. Red arrow: Mediastinal lymph node enlargement; black arrow: The point for percutaneous lung biopsy. CT: computed tomography.

**图 2:**
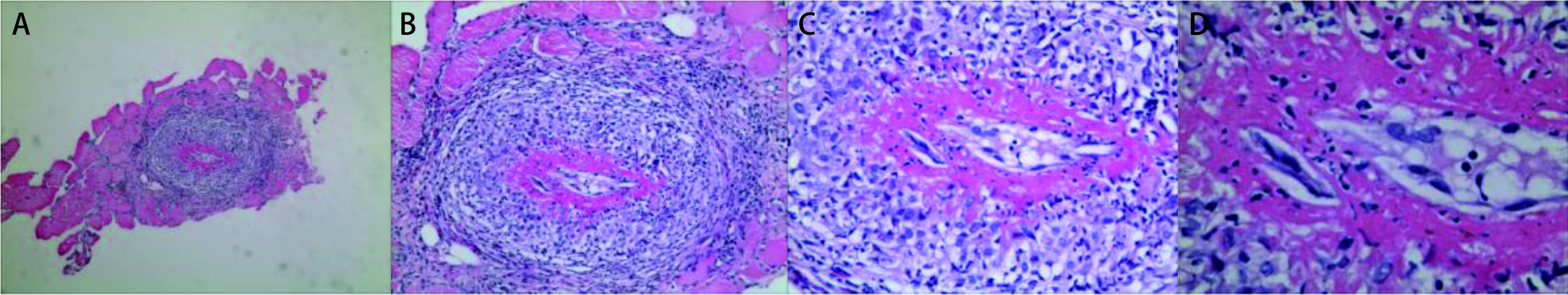
不同倍镜下的HE染色。A：40×；B：100×；C：200×；C：400×。组织标本的病理分析。肺穿刺组织HE染色可见肺组织、纤维结缔组织及骨骼肌组织，肺组织示慢性炎症，肺泡间隔内可见淋巴细胞、浆细胞及单核细胞浸润，余骨骼肌组织中见小血管、血管壁纤维素样坏死伴中性粒细胞渗出，血管周围上皮细胞肉芽肿形成，结合临床，考虑韦格纳肉芽肿可能。 Photo of HE staining under different magnification. A: 40×; B: 100×; C: 200×; C: 400×. The analysis of the pathology. HE staining of lung puncture tissue showed lung tissue, fibrous connective tissue and skeletal muscle tissue. Lung tissue shows chronic inflammation, infiltration of lymphocytes, plasma cells and monocytes can be seen in alveolar septum, cellulose like necrosis of small blood vessels and blood vessel wall with neutrophil exudation can be seen in other skeletal muscle tissues, and perivascular epithelial cell granuloma can be formed. Combined with clinical practice, Wegener granuloma may be considered.

**图 3:**
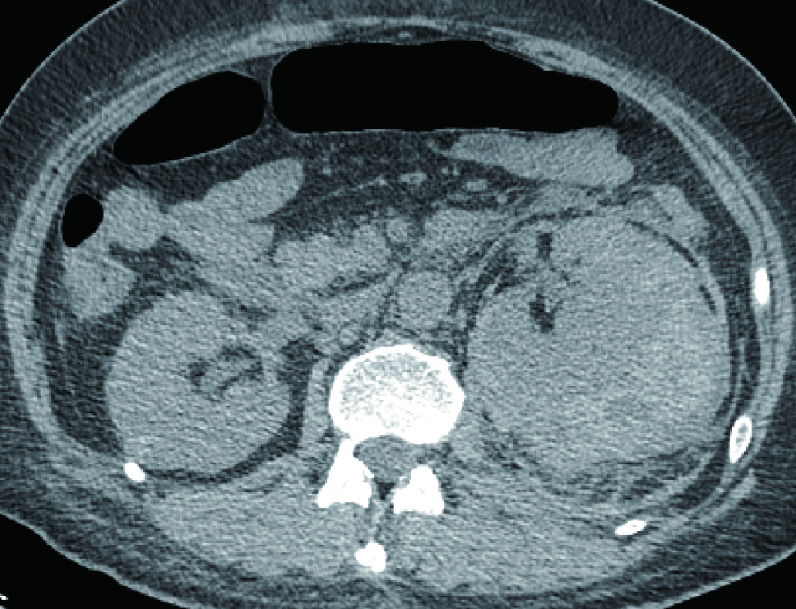
腹部CT示左侧肾筋膜内巨大血肿 CT scans of the abdomen shows large hematoma within the left renal fascia

## 2 讨论

WG以肺肉芽肿变为基本表现，同时可伴有典型的上呼吸道和肾脏损害，伴有肾脏损害者多见，无肾脏损害者称为局限型GPA。WG作为自身免疫缺陷疾病，全身器官均可受累，临床曾有报道有首发症状表现为胸骨骨髓炎^[3]^、缩窄性心包炎^[4]^及较为少见的视网膜闭塞性血管炎^[5]^。本例患者表现为较为典型的肺部受累症状，患者无明显诱因出现咳嗽咳痰，胸CT提示右肺门肿物，伴双肺门及纵隔多发淋巴结肿大，同时肾脏损害进展迅速，1周内即迅速出现血尿及蛋白尿。

由于目前尚无WG诊断的金标准，国内外仍应用1990年美国风湿病学会（American College of Rheumatology, ACR）分类标准协助诊断：①鼻或口腔炎症痛性或无痛性口腔溃疡，脓性或血性鼻腔分泌物；②胸部影像学异常，胸片示结节、固定浸润病灶或空洞；③尿沉渣异常镜下血尿（红细胞 > 5个/高倍视野），或出现红细胞管型；④病理性肉芽肿性炎性改变，动脉壁或动脉周围或血管（动脉或微动脉）外区有中性粒细胞浸润。在以上4条表现中符合2条或2条以上时可诊断为WG，诊断的敏感性和特异性分别为88.2%和92.0%。而WG作为病情发展迅速的自身免疫性疾病，未得到早期诊疗的患者死亡率较高，因此早期确诊WG对于患者良好预后具有重大意义。

WG是ANCA相关性血管炎（ANCA-associated vasculitidies, AAV）中的一个亚型。ANCA指标对AAV具有重要诊断价值，其平均敏感度范围为66%（95%CI: 57%-74%），平均特异性范围高达98%（95%CI: 96%-99.5%）^[6]^。除WG外，AAV还包括显微镜下多血管炎（multiple vasculitis, MPA）、变应性肉芽肿性血管炎（allergic granulomatous vasculitis, EGPA）及Churg-Strauss综合征（Churg-Strauss syndrome, CSS），应在临床诊疗中相互鉴别。WG主要见C-ANCA阳性，病理学表现以累及上下呼吸道的肉芽肿性病变为主要特征，多累及中型血管；MPA则表现为P-ANCA和MPO阳性，常见坏死性血管炎，肾小球性肾炎及毛细血管炎，少见肉芽肿性病变^[6, 7]^。EGPA中上呼吸道损害较为少见，CSS则表现为重度哮喘及血嗜酸性粒细胞增高，临床上较容易区分。

尽管ANCA相关抗体对于WG的诊断具有较高价值，仍有其他结缔组织病（如系统性红斑狼疮、风湿性关节炎、进行性系统性硬化病等）也可见ANCA相关抗体的升高。因此，为了及时对WG患者提供诊疗方案，除免疫学指标外，综合影像学检查以及组织活检以进行早期诊断是十分必要的^[8]^。

WG的CT表现为团块影、斑片影等非特异性改变，与感染性及肿瘤性病变均难以区分。在正电子发射型计算机断层显像（positron emission computed tomography, PET）/CT中肿瘤与WG均表现为高代谢状态，因此仅能排除低代谢的的炎性包块，但其优势在于可以准确定位病变位置，便于后续活检。另外在已确诊病例的治疗过程中，PET/CT可以很好地判断治疗效果^[9]^。

组织活检是WG早期诊断的决定性依据，气管镜活检作为常见的肺部疾病检查手段，具有创伤小、取材可靠的优点^[10]^，但气管镜检查受气道限制，对外周肉芽肿性病变取材困难，使得其在WG诊断中的应用价值十分有限；而CT引导下经皮肺穿刺作为一种取材范围广泛的检查手段，其对于外周的病变取材的准确性高，在WG早期诊断中具有明显优势。因此气管镜活检结合CT引导下经皮肺穿刺，基本可以满足包括WG早期诊断在内的大部分临床诊疗需求。

随着对AAV疾病的认识加深以及免疫疗法的介入，WG已经从1年死亡率近80%的危重症^[2]^发展为5年生存率高达75%的慢性复发性疾病^[11, 12]^。激素疗法及以环磷酰胺为主的免疫抑制疗法为WG的主要治疗手段。此外还有仍处于研究阶段生物制剂疗法，可通过清除B细胞达到治疗目的。研究^[13]^表明利妥昔单抗与环磷酰胺相比，在疗效上没有太大优势，而且早期的严重不良反应事件也没有减少，但其在复发病例中更有潜在优势。英夫利昔单抗和阿达木单抗与固醇激发剂疗效相当，而依那西普单抗则效果不佳^[14]^。此外，对于激素及免疫抑制治疗效果不佳且病情危重的患者，可以考虑血浆置换治疗^[15]^。

本例患者以非特异性呼吸道损害为首发临床表现，伴有急性肾损伤与多器官受累，属于较为典型的WG表现，免疫学检查示ANCA-C型-IIF及ANCA-P型-IIF阳性，CT引导下经皮肺穿刺示血管周围上皮细胞肉芽肿，考虑WG可能，遂转入专科进行后续治疗。由于早期诊断，患者及时得到了有效治疗，2周后顺利出院，预后良好。由此个案可提示，结合临床症状、血免疫学检查、影像学检查及病理学检查可对WG做出早期明确诊断，为患者争取最佳治疗时机。

## References

[1] Chen XH (2018). Big data analysis of misdiagnosis in China (Volume Ⅰ). Nanjing: Southeast University Press.

[2] Walton EW (1958). Giant-cell granuloma of the respiratory tract (Wegener's granulomatosis). Br Med J.

[3] Kim SD, Kim GW, Kim TE (2013). Granulomatosis with polyangiitis (Wegener granulomatosis) as a differential diagnosis of sternal osteomyelitis: the challenges in diagnosis. J Clin Rheumatol.

[4] Horne AE, Henriksen PA, Amft EN (2014). Granulomatosis with polyangiitis and constrictive pericarditis-a case report. J R Coll Physicians Edinb.

[5] Hachicha F, Brour J, Zahaf A (2014). Progression of a rare and serious ocular manifestation of Wegener's granulomatosis: Occlusive retinal vasculitis. J Fr Ophtalmol.

[6] Jaya K., Rao MD, Morris Weinberger P (2015). The role of antineutrophil cytoplasmic antibody (c-ANCA) testing in the diagnosis of Wegener granulomatosis. Ann Inter Med.

[7] Jennette JC, Falk RJ, Bacon PA (2013). 2012 revised International Chapel Hill Consensus Conference Nomenclature of Vasculitides. Arthritis Rheum.

[8] Gal AA, Velasquez A (2002). Antineutrophil cytoplasmic autoantibody in the absence of Wegener's granulomatosis or microscopic polyangiitis: implications for the surgical pathologist. Mod Pathol.

[9] Yamashita H, Kubota K, Mimori A Clinical value of whole-body PET/CT in patients with active rheumatic diseases. Arthritis Res Ther.

[10] Bose S, Ghatol A, Eberlein M (2013). Ultrathin bronchoscopy in the diagnosis of peripheral cavitary lung lesions. J Bronchology Interv Pulmonol.

[11] Booth AD, Almond MK, Burns A (2003). Outcome of ANCA-associated renal vasculitis: a 5-year retrospective study. Am J Kidney Dis.

[12] Flossmann O, Berden A, de Groot K (2011). Long-term patient survival in ANCA-associated vasculitis. Ann Rheum Dis.

[13] Jones RB, Tervaert JW, Hauser T (2010). Rituximab versus cyclophosphamide in ANCA-associated renal vasculitis. N Engl J Med.

[14] Silva-Fernandez L, Loza E, Martinez-Taboada VM (2014). Biological therapy for systemic vasculitis: a systematic review. Semin Arthritis Rheum.

[15] Malhotra S, Dhawan HK, Sharma RR (2016). Successful management of refractory dialysis independent Wegener's granulomatosis with combination of therapeutic plasma exchange and rituximab. Indian J Hematol Blood Transfus.

